# Screening the Cancer Genome Atlas Database for Genes of Prognostic Value in Acute Myeloid Leukemia

**DOI:** 10.3389/fonc.2019.01509

**Published:** 2020-01-21

**Authors:** Jie Ni, Yang Wu, Feng Qi, Xiao Li, Shaorong Yu, Siwen Liu, Jifeng Feng, Yuxiao Zheng

**Affiliations:** ^1^Department of Medical Oncology, Jiangsu Cancer Hospital, Jiangsu Institute of Cancer Research, The Affiliated Cancer Hospital of Nanjing Medical University, Nanjing, China; ^2^Research Center for Clinical Oncology, Jiangsu Cancer Hospital, Jiangsu Institute of Cancer Research, The Affiliated Cancer Hospital of Nanjing Medical University, Nanjing, China; ^3^Department of Urology, The First Affiliated Hospital of Nanjing Medical University, Nanjing, China; ^4^Department of Urology, Jiangsu Cancer Hospital, Jiangsu Institute of Cancer Research, The Affiliated Cancer Hospital of Nanjing Medical University, Nanjing, China

**Keywords:** immune/stromal scores, tumor microenvironment (TME), biomarkers, immune infiltrates, acute myeloid leukemia (AML)

## Abstract

**Object:** To identify genes of prognostic value which associated with tumor microenvironment (TME) in acute myeloid leukemia (AML).

**Methods and Materials:** Level 3 AML patients gene transcriptome profiles were downloaded from The Cancer Genome Atlas (TCGA) database. Clinical characteristics and survival data were extracted from the Genomic Data Commons (GDC) tool. Then, limma package was utilized for normalization processing. ESTIMATE algorithm was used for calculating immune, stromal and ESTIMATE scores. We examined the distribution of these scores in Cancer and Acute Leukemia Group B (CALGB) cytogenetics risk category. Kaplan-Meier (K-M) curves were used to evaluate the relationship between immune scores, stromal scores, ESTIMATE scores and overall survival. We performed clustering analysis and screened differential expressed genes (DEGs) by using heatmaps, volcano plots and Venn plots. After pathway enrichment analysis and gene set enrichment analysis (GESA), protein-protein interaction (PPI) network was constructed and hub genes were screened. We explore the prognostic value of hub genes by calculating risk scores (RS) and processing survival analysis. Finally, we verified the expression level, association of overall survival and gene interactions of hub genes in the Vizome database.

**Results:** We enrolled 173 AML samples from TCGA database in our study. Higher immune score was associated with higher risk rating in CALGB cytogenetics risk category (*P* = 0.0396) and worse overall survival outcomes (*P* = 0.0224). In Venn plots, 827 intersect genes were screened with differential analysis. Functional enrichment clustering analysis revealed a significant association between intersect genes and the immune response. After PPI network, 18 TME-related hub genes were identified. RS was calculated and the survival analysis results revealed that high RS was related with poor overall survival (*P* < 0.0001). Besides, the survival receiver operating characteristic curve (ROC) showed superior predictive accuracy (area under the curve = 0.725). Finally, the heatmap from Vizome database demonstrated that 18 hub genes showed high expression in patient samples.

**Conclusion:** We identified 18 TME-related genes which significantly associated with overall survival in AML patients from TCGA database.

## Introduction

Acute myeloid leukemia (AML) is a hematopoietic clonal malignancy characterized by uncontrolled proliferation of hematopoietic stem cells (HSCs) and progenitor cells without the ability to differentiate into mature cells (1). The treatment and prognosis of patients with AML depend on accurate cytogenetics and genetic testing (2). Recently, significant progress has been made in the basic and preclinical studies of acute myeloid leukemia (AML). The improvement in AML is largely due to advances in supportive care and hematopoietic cell transplantation rather than conventional chemotherapy. However, due to the high recurrence rate, the 5-year survival rate is still very low, so there is an urgent need for novel and effective treatment methods (3). More and more attention has been focused on identifying appropriate AML immunotherapy strategies.

Since immune checkpoint therapies such as CTLA-4 (4) and PD-1 (5) have developed rapidly in AML in recent years, tumor microenvironment (TME) is an important cellular environment for immune cells, stromal cells, and extracellular matrix molecules and has attracted more and more attention (6, 7).

TME is a cellular environment in which tumor lesions are present. It consists of endothelial cells, inflammatory mediators, mesenchymal cells, and immune and stromal cells (8, 9). Among them, immune cells and stromal cells are two major non-tumor components, which are of great significance in the diagnosis and prognosis of cancer. AML tumor cells form a complex environment of the tumor microenvironment, which ultimately promotes the adaptability and disease progression of tumor cell transcriptome (10). On the other hand, TME has been found to have a severe effect on gene expression in cancer tissues therefore affecting clinical outcomes (11–16).

To further investigate the molecular biological properties of TME, algorithms for gene expression data using The Cancer Genome Atlas (TCGA) database have been developed. The TCGA database is a complete genome-wide gene expression profile for categorizing and detecting genomic abnormalities in a large population worldwide (14, 17–19). For example, Yoshihara et al. designed an algorithm called ESTIMATE that uses expression data to estimate stromal cells and immune cells in malignant tumors (14). In this algorithm, the expression characteristics of specific genes in immune cells and stromal cells are analyzed to calculate immune and stromal score to predict non-tumor cell invasion. Recent reports indicate that ESTIMATE is used in the study of prostate cancer (20), breast cancer (21), and colon cancer (22). However, the characteristics of the TME evaluated by ESTIMATE were not observed in the AML.

To obtain more insights, we extracted the list of microenvironment-related genes that predicted poor prognosis of AML patients by using the TCGA database of AML cohort and the immune score derived from the ESTIMATE algorithm (14). More importantly, we developed a risk scoring system to evaluate the prognostic value of central genes. In addition, the correlation between central gene and immune infiltration was also discussed.

## Methods And Materials

### Data Collection

Level three gene transcriptome profiles of AML patients in The Cancer Genome Atlas (TCGA) database (https://portal.gdc.cancer.gov/) were collected. We enrolled sample data ended with “−03” in sample codes, which means that these data belong to the “Primary Blood Derived Cancer-Peripheral Blood.” RNA expression for AML Multiforme was obtained from IlluminaHiSeq (version: 2017-10-13). After that, we downloaded the survival data through the Genomic Data Commons (GDC) tool from TCGA. Sex, Cancer and Acute Leukemia Group B (CALGB) cytogenetics risk category and survival condition were extracted. We excluded AML samples that did not end in “−03” and samples with incomplete survival and clinical information. We used limma package for normalization processing (23). Scores of immune, stromal and ESTIMATE were calculated using ESTIMATE algorithm (https://sourceforge.net/projects/estimateproject/).

### Correlation Analysis and Survival Analysis

Ordinary one-way analysis of variance was performed to show the association between immune scores, stromal scores, ESTIMATE scores, and the CALGB cytogenetics risk category. Kaplan-Meier (K-M) curves with log-rank test was based on survival package (24, 25). We used K-M curves to evaluate the relationship between immune scores, stromal scores, ESTIMATE scores, and overall survival. *P* < 0.05 was considered as statistically significant.

### Heatmaps, Clustering Analysis, and Differentially Expressed Genes

We divided the immune scores and the stromal scores into high and low groups by median. We set |log(FC)| >1 and false discovery rate (FDR) <0.05 as standard of limma package which used for standardization of transcriptome data (23). To express the results of differentially expressed gene (DEG) screening and cluster analysis, |log(FC)| >1 and FDR <0.05 were set in performing heatmaps; cut |log2FC| = 1 and cut *P* = 0.05 were set in performing volcano plots based on a pheatmap package, ggplot2 package, and clustering analysis. After that, intersected DEGs were screened among immune scores and stromal scores by Venn plots based on VennDiagram package (26).

### Enrichment Analysis of Differentially Expressed Genes and Gene Set Enrichment Analysis

The Database for Annotation, Visualization, and Integrated Discovery (DAVID, https://david.ncifcrf.gov/) was used for the construction of gene ontology (GO) analysis by biological processes (BP), cellular components (CC), and molecular functions (MF) (27). In addition, the Kyoto Encyclopedia of Genes and Genomes (KEGG) analysis with *q* < 0.05 was performed based on org.Hs.eg.db package, clusterProfiler, org.Hs.eg.db, enrichplot, and ggplot2 packages. In the gene set enrichment analysis (GSEA) with FDR <0.25, |enriched score|> 0.35, and gene size ≥35, we selected “c2.cp.kegg.v6.2.symbols.gmt gene sets” as gene set database and “Illumina_Human.chip” as chip platform (28).

### Protein-Protein Interaction Network and Hub Genes

Protein-protein interaction (PPI) network construction with minimum required interaction score = 0.9 was based on the STRING database (version 11.0) and Cytoscape software (version 3.7.1) (29, 30). We used cytoHubba to identify hub genes (31). In cytoHubba, we selected top 10 nodes from each of the 12 algorithms, and the genes with degree <10 were ruled out.

### Survival Curve and Risk Score

After hub genes were detected, we evaluated the prognostic value by K-M analysis based on log-rank test. *P* < 0.05 was regarded as statistically significant. Risk score (RS), which statistically equals to Σ (β_i_^*^ Exp_i_) (*i* = the number of prognostic hub genes), was calculated for every AML patients based on multivariate Cox regression analysis. Then, patients were separated into high- and low-risk groups according to the median RS. In addition, K-M curves were used to explore the association between different RS level and overall survival. The survival receiver operating characteristic curve (ROC) was drawn and the area under the curve (AUC) was calculated for evaluating prognostic value (32).

### Vizome Database Analysis

Vizome is the largest AML database which contains the whole-exome sequencing of a cohort of 672 tumor specimens collected from 562 patients (33). We verified the expression level, association of overall survival, and gene interactions of hub genes in the vizome database.

### Statistical Analysis

IBM SPSS Statistics 20.0 was applied in multivariate Cox regression analysis and K-M analysis. R software (version 3.5.2) was utilized for statistical analysis. *P* < 0.05 represented as statistically significant.

The flow diagram representing the work was shown in Supplementary Figure 3.

## Results

### Immune Score Was Associated With Cancer and Acute Leukemia Group B Cytogenetics Risk Category and Survival Outcome

**Figure 1:**
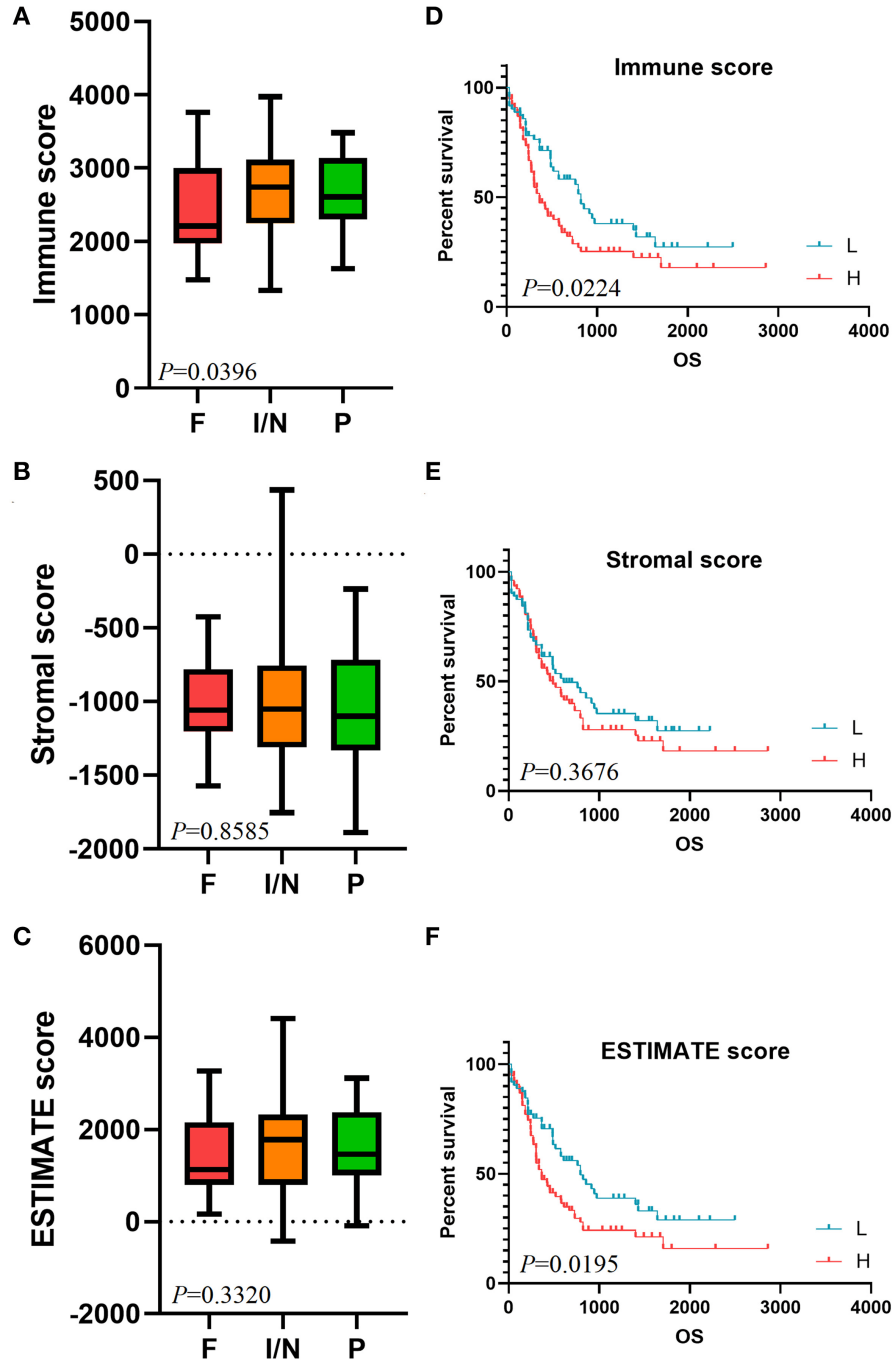
Immune score is associated with CALGB cytogenetics risk category and survival outcome. The distribution of immune scores **(A)**, stromal scores **(B)**, and ESTIMATE scores **(C)** in CALGB cytogenetics risk category were shown in box plots. K-M survival curves in immune score group **(D)**, stromal score group **(E)**, and ESTIMATE score group **(F)** revealed the relationships between different score levels and overall survival. CALGB, Cancer and Acute Leukemia Group B; F, favorable; I/N, intermediate/normal; P, poor; K-M, Kaplan-Meier; L, low score group; H, high score group.

We enrolled 173 AML samples from TCGA database with 93 males (53.76%) and 80 females (46.24%) in our study. Clinical characteristics of AML patients were listed in Table 1. Besides, we performed immune scores, stromal scores, and ESTIMATE scores in Supplementary Table 1. Box plots revealed that higher immune score was associated with a higher risk rating in CALGB cytogenetics risk category (*P* = 0.0396, Figure 1A). However, significant results were not observed based on stromal scores and ESTIMATE scores (*P* = 0.8585 and *P* = 0.3320, respectively; Figures 1B,C). Then, we divided AML samples into high-score groups and low-score groups according to the median of immune scores, stromal scores, and ESTIMATE scores, respectively. K-M curves were performed to evaluate the relationships between different score levels and overall survival. The results revealed that higher immune score and ESTIMATE score were associated with worse overall survival outcomes (*P* = 0.0224, *P* = 0.0195, respectively; Figures 1D,F), whereas no significant results were found in stromal scores group (*P* = 0.3676, Figure 1E).

**Table 1:** Clinical Characteristics of 173 AML patients from TCGA cohort.

	**Male (%)**	**Female (%)**	**Total (%)**
Number	93 (53.76)	80 (46.24)	173 (100)
CALGB			
Favorable	16 (9.25)	16 (9.25)	32 (18.50)
Intermediate/Normal	52 (30.06)	51 (29.48)	103 (59.54)
Poor	25 (14.45)	11 (6.36)	36 (20.81)
Not Available	0 (0)	2 (1.16)	2 (1.16)
Event			
Dead	56 (32.37)	49 (28.32)	105 (60.69)
Alive	32 (18.50)	26 (15.03)	58 (33.53)
Not Available	5 (2.89)	5 (2.89)	10 (5.78)

### Comparison of Gene Expression Profiles With Immune Scores and Stromal Scores in Acute Myeloid Leukemia

**Figure 2:**
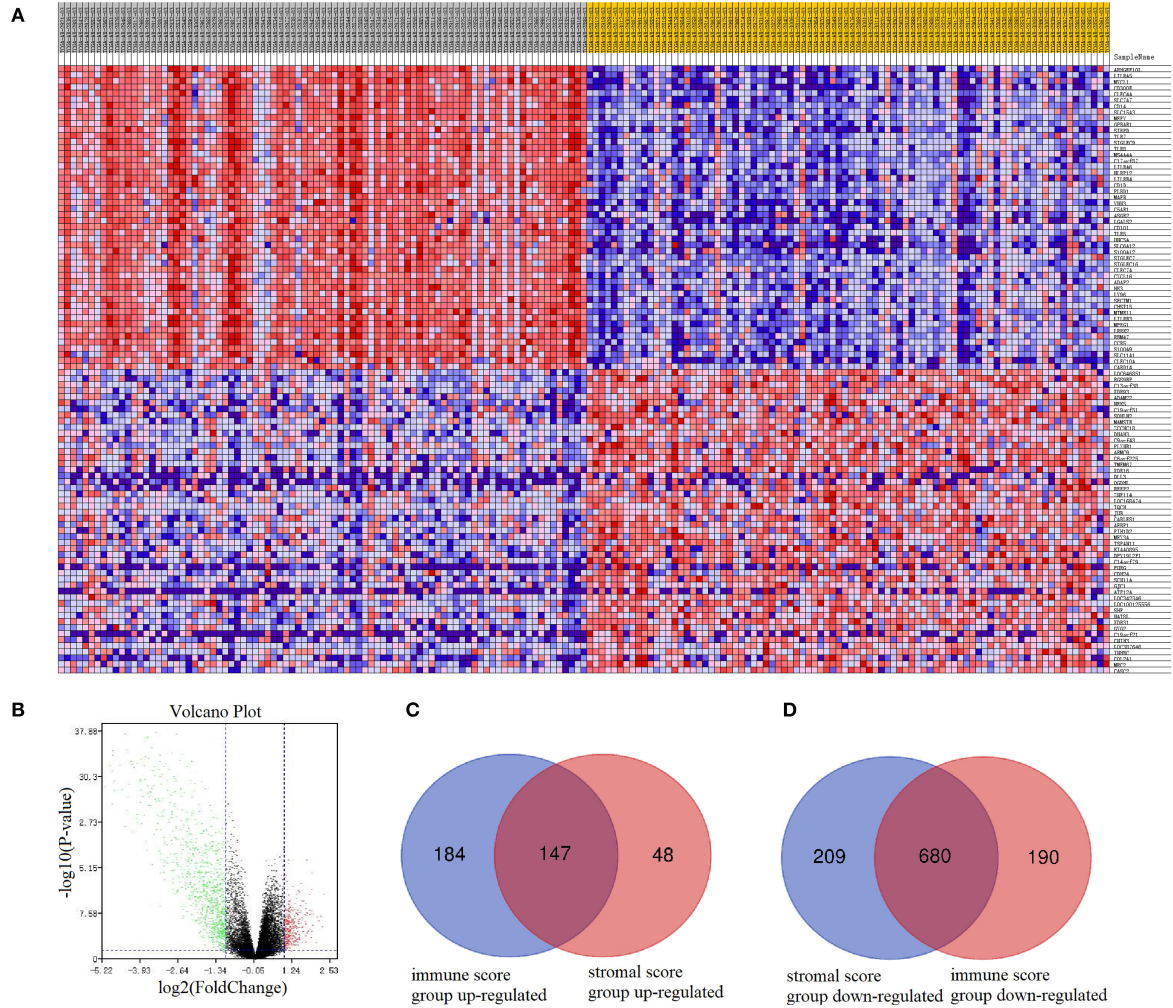
Comparison of gene expression profiles with immune scores and stromal scores in AML. In immune score group, heatmap **(A)** and volcano plot **(B)** were used to demonstrated differential expressed genes. Venn plots were performed to reveal up-regulated intersect genes **(C)** and down-regulated intersect genes **(D)**.

We constructed a heatmap of clustering analysis in Figure 2A. The right side of the samples was the low immune score group, while the left half was the high immune score group. Besides, DEGs based on the immune score group were reflected in volcano plot (Figure 2B). In the stromal score group, the heatmap and volcano plot were shown in Supplementary Figure 1. Furthermore, we screened 331 up-regulated DEGs and 889 down-regulated DEGs in the immune score group (Supplementary Table 2) and screened 195 up-regulated DEGs and 870 down-regulated DEGs in the stromal score group (Supplementary Table 3). In the Venn plots, 147 up-regulated intersected genes (Figure 2C) and 680 down-regulated intersected genes were screened (Figure 2D).

### Functional Enrichment Analysis

**Figure 3:**
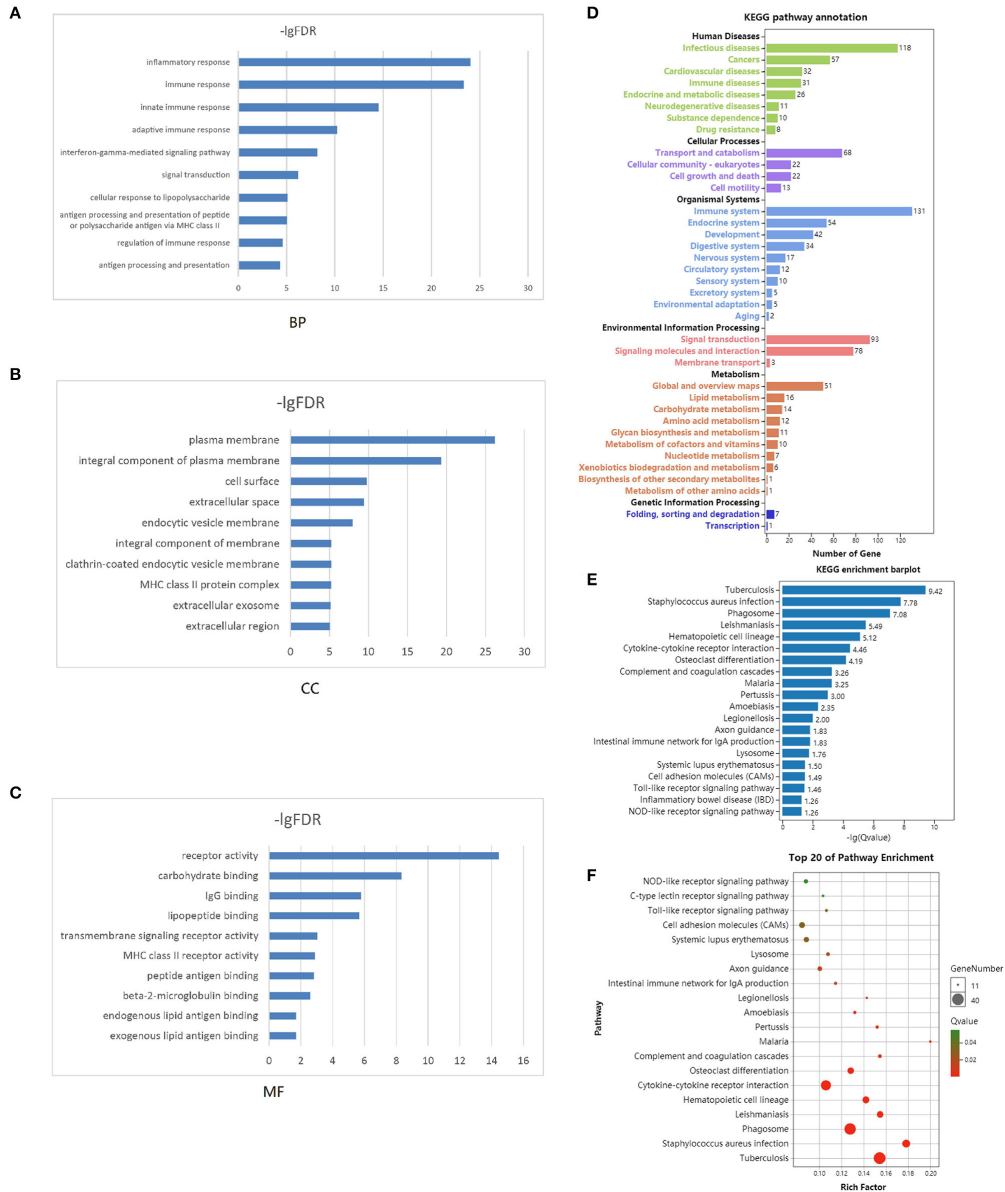
Functional enrichment analysis. Top 10 GO terms in each of biological process **(A)**, cellular component **(B)**, and molecular function **(C)** were performed for functional enrichment clustering analysis. KEGG pathway analysis were performed as pathway annotation **(D)**, enrichment barplot **(E)**, and bubble chart of top 20 pathway enrichment analysis **(F)**. GO, gene ontology; BP, biological processes; CC, cellular components; MF, molecular functions; KEGG, Kyoto Encyclopedia of Genes and Genomes.

Functional enrichment clustering analysis revealed a significant association between intersected genes and the immune response. We selected top 10 GO terms in each of the biological process (Figure 3A), cellular component (Figure 3B), and molecular function (Figure 3C). Inflammatory response, immune response, plasma membrane, receptor activity were top GO terms identified in our analysis. In the KEGG pathway annotation (Figure 3D) and enrichment analysis (Figure 3E), we found pathways associated with immunity, cancer, and tuberculosis. The top 20 pathway enrichment analysis was shown in Figure 3F, where bubble size represented gene number and color represented Q value.

### Protein-Protein Interaction and Hub Gene Identification

**Figure 4:**
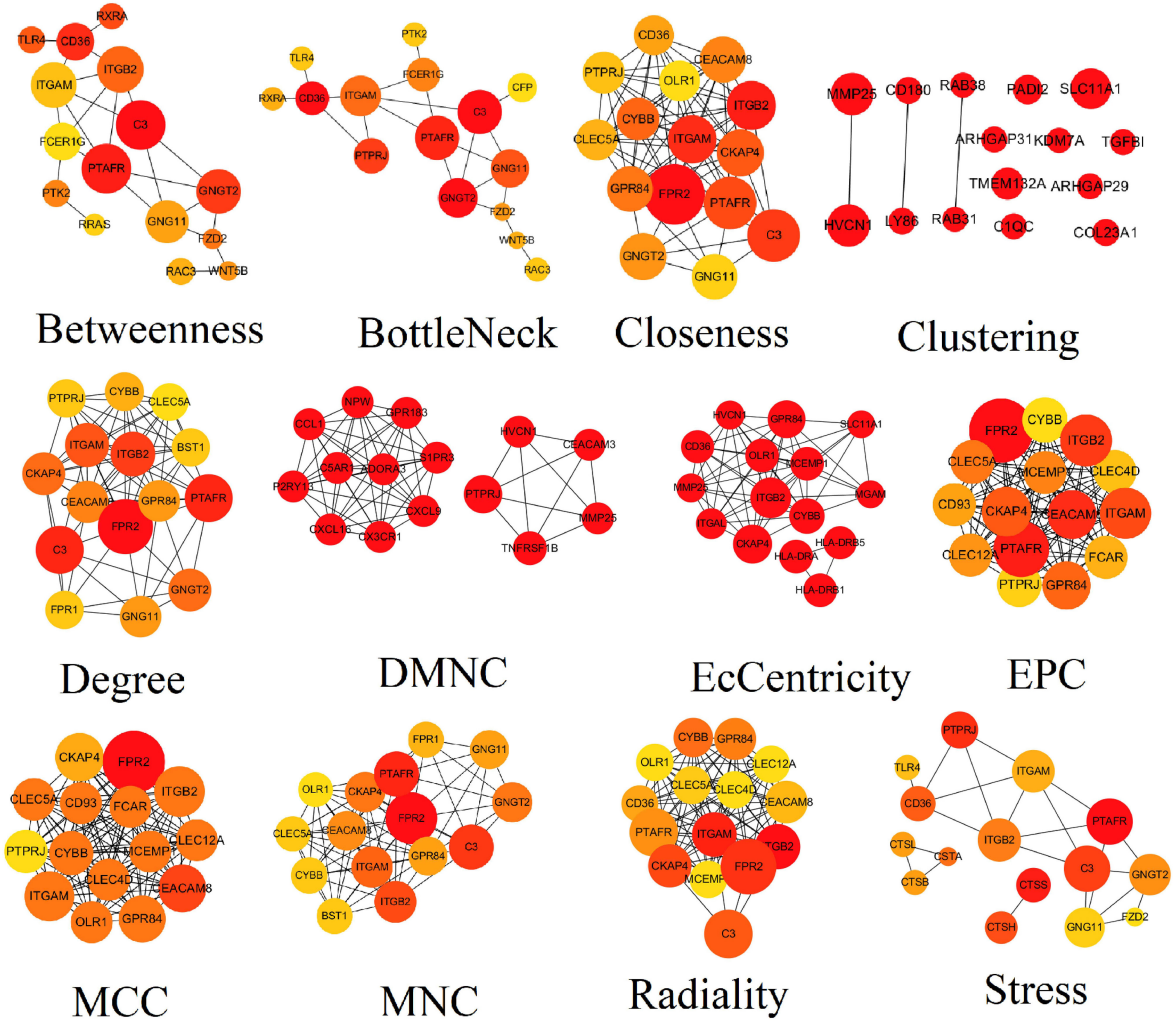
Results of algorithms from cytoHubba. The PPI network data from STRING was further analyzed by Cytoscape and hub genes identification was performed by cytoHubba based on 12 algorithms. PPI, protein-protein interaction.

PPI network contained 786 nodes and 1,774 edges. Results from STRING were further analyzed by Cytoscape. The results of algorithms from cytoHubba applied in hub gene identification were shown in Figure 4. Circle size was represented by node degree. Finally, 18 TME-related hub genes were identified as follows: ITGAL, ITGAM, HLA-DRB1, HLA-DRB5, FPR1, CX3CR1, TNFRSF1B, CXCL16, CTSB, CTSS, HLA-DRA, P2RY13, ITGB2, CEACAM3, SLC11A1, C5AR1, ADORA3, and GNGT2.

### Gene Set Enrichment Analysis

**Figure 5:**
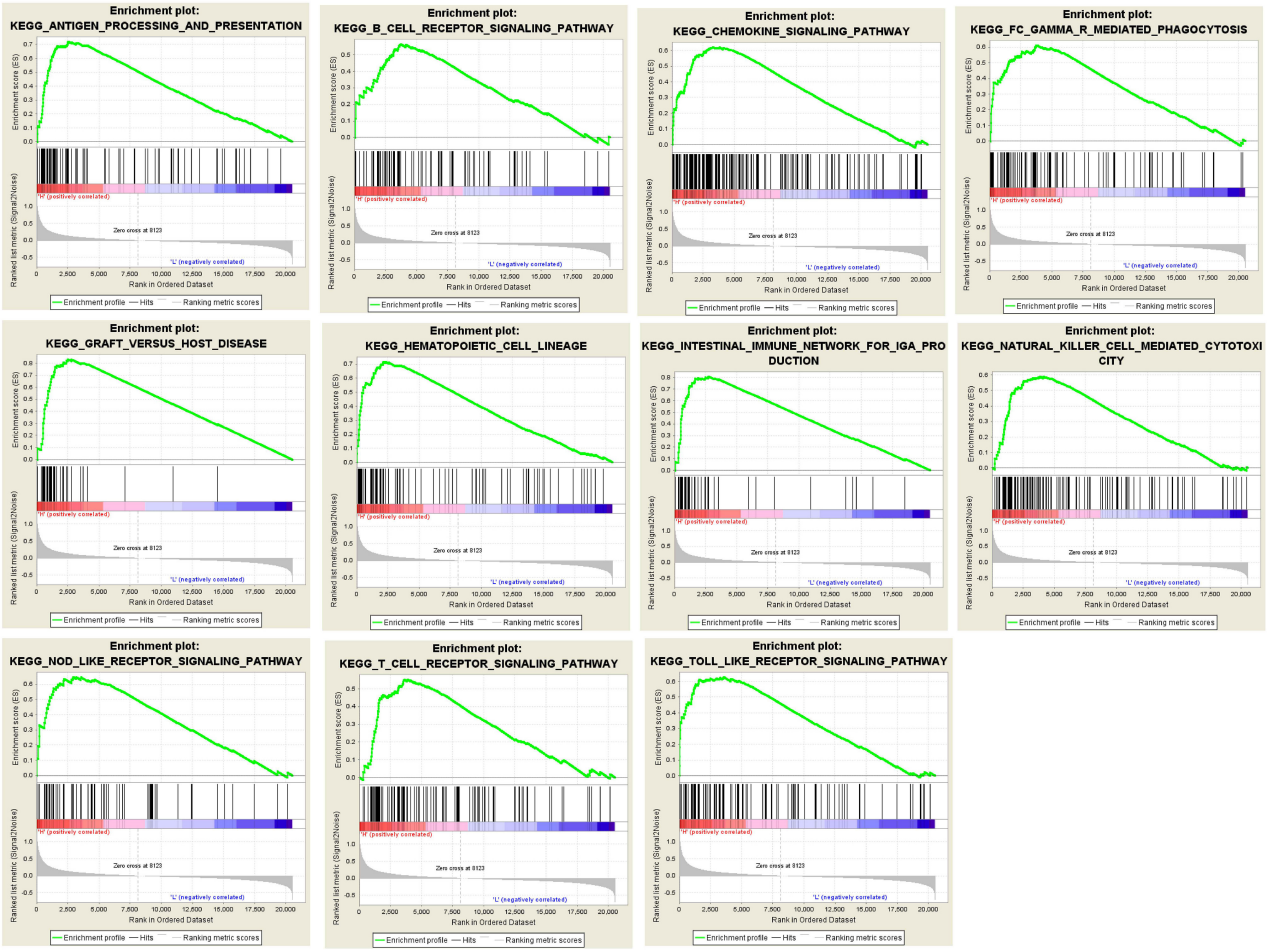
Results of GSEA analysis. GSEA analysis was performed to further screen the significant pathway between higher immune scores group and lower immune scores group. GSEA, gene set enrichment analysis.

The results of GSEA revealed that antigen processing and presentation, B cell receptor signaling pathway, chemokine signaling pathway, FcγR mediated phagocytosis, graft vs. host disease, hematopoietic cell lineage, intestinal immune network for IgA production, natural killer cell mediated cytotoxicity, nucleotide binding oligomerization domain (NOD) like receptor signaling pathway, T cell receptor signaling pathway, and Toll like receptor signaling pathway were main pathways enriched by intersected genes related to tumor immunity (Figure 5).

### Risk Score and Survival Analysis

**Figure 6:**
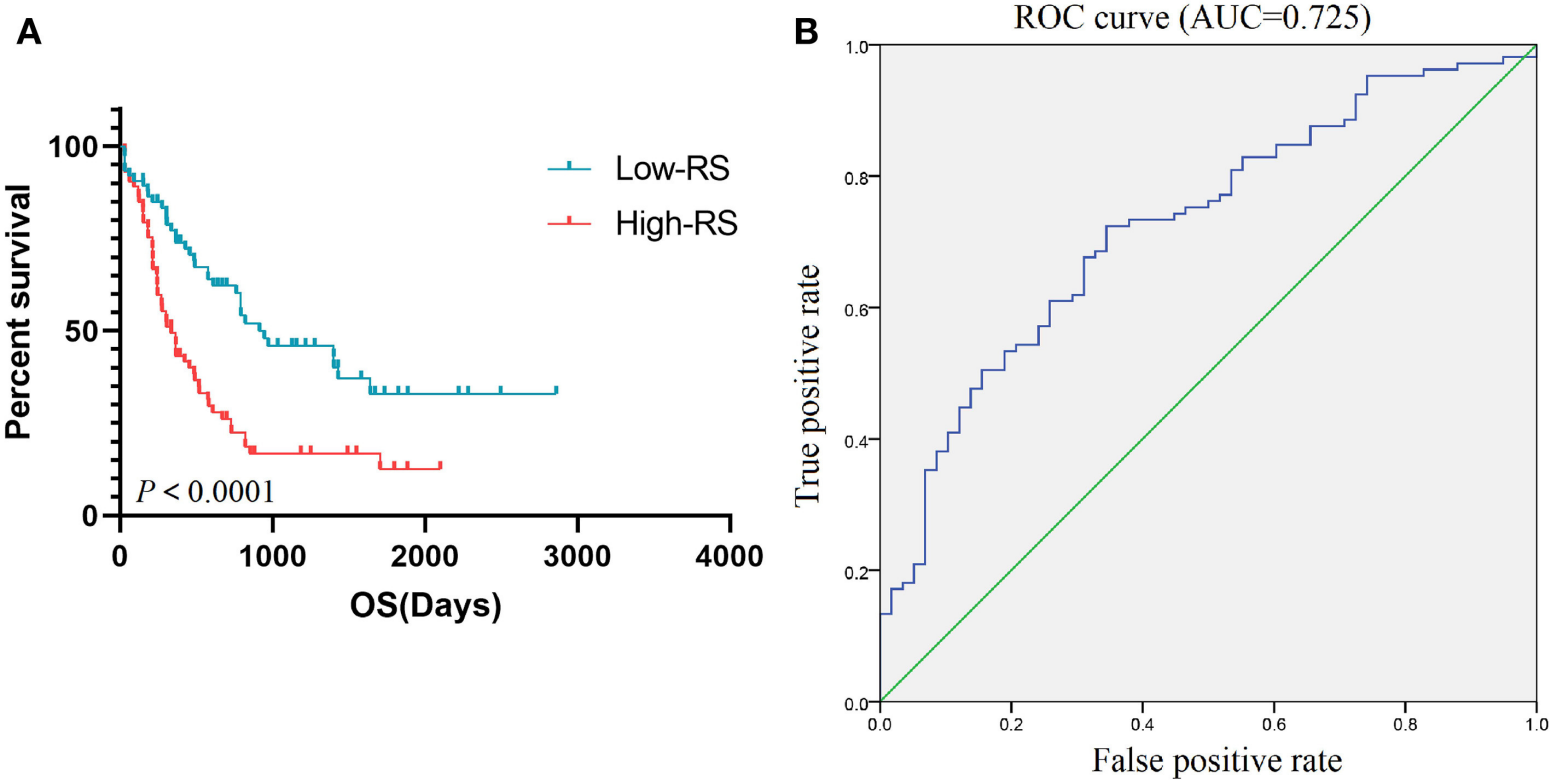
Prognostic value of RS. K-M curve **(A)** based on low-RS group and high-RS group and ROC curve **(B)** with AUC = 0.725 represented the prognostic value of RS. K-M, Kaplan-Meier; RS, risk score; OS, overall survival; ROC, operating characteristic curve; AUC, area under the curve.

**Figure 7:**
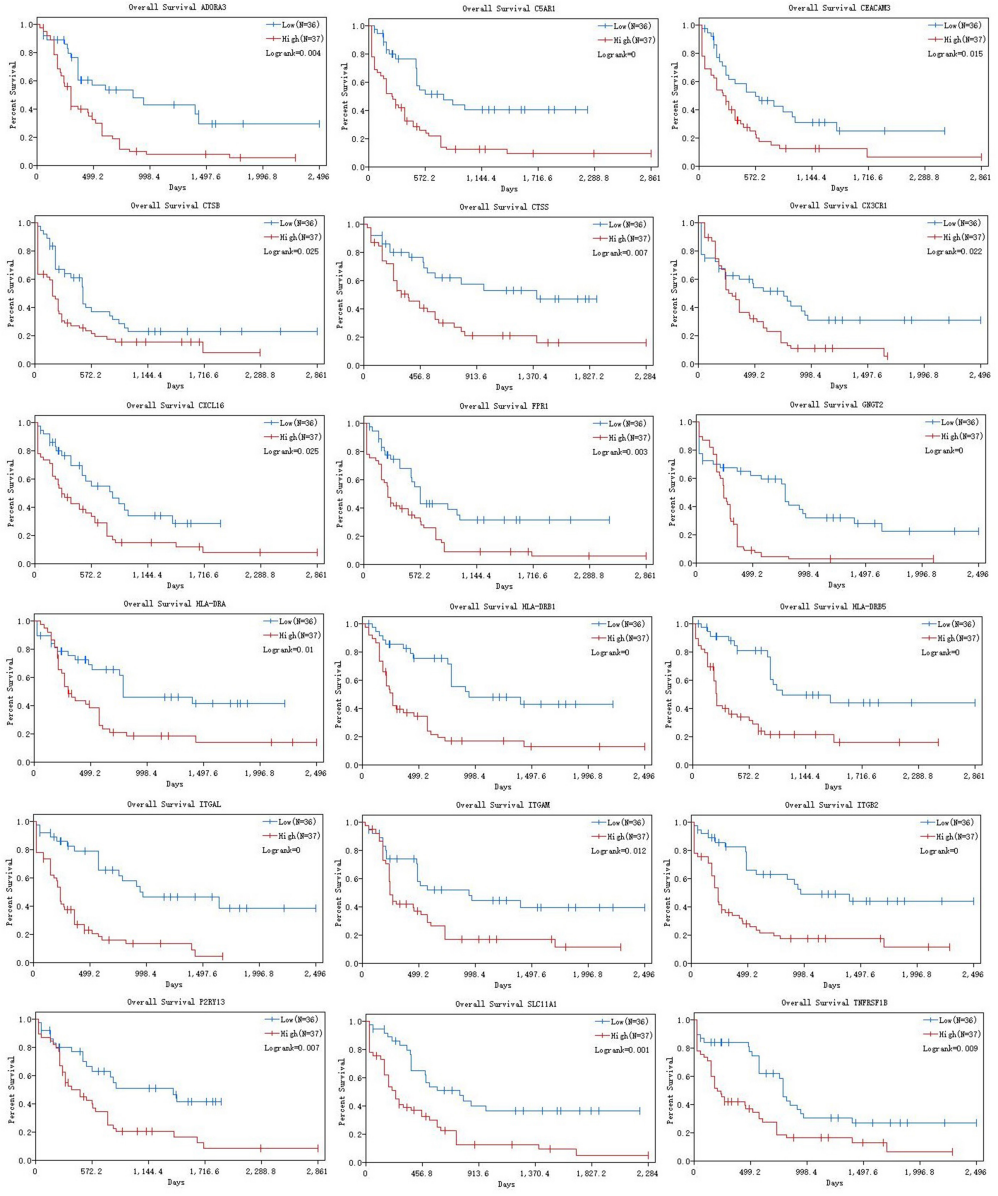
K-M curves of 18 hub genes. K-M curves were applied to explore the association between expression levels of hub genes and overall survival. K-M, Kaplan-Meier.

According to the results of the Cox regression analysis, the RS calculation formula is as follows: RS = ITGAL ^*^ 0.177 + ITGAM ^*^ 0.315 + HLA-DRB1 ^*^ 0.371 + HLA-DRB5 ^*^ (−0.009) + FPR1 ^*^ 0.034 + CX3CR1 ^*^ (−0.074) + TNFRSF1B ^*^ 0.172 + CXCL16 ^*^ (−0.104) + CTSB ^*^ (−0.38) + CTSS ^*^ (−0.201) + HLA-DRA ^*^ (−0.353) + P2RY13 ^*^ 0.003 + ITGB2 ^*^ 0.038 + CEACAM3 ^*^ (−0.051) + SLC11A1 ^*^ (−0.034) + C5AR1 ^*^ (−0.049) + ADORA3 ^*^ 0.213 + GNGT2 ^*^ 0.208. We divided 163 eligible AML patients into low-RS group and high-RS group according to the median. The result of survival analysis demonstrated that high RS was related with poor overall survival (Figure 6A). To evaluate the prognostic value of RS, we drew the ROC curve and calculated the AUC. From Figure 6B, AUC was 0.725, which revealed superior predictive accuracy in overall survival. Besides, we constructed survival curves of 18 hub genes for exploring prognostic value (Figure 7). The results showed that the high expression level of hub genes was associated with poor overall survival.

### Vizome Database Analysis

**Figure 8:**
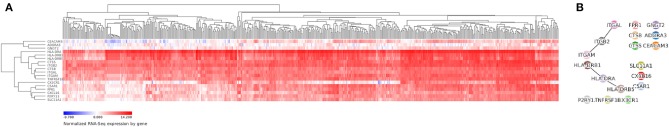
Vizome database analysis. We verified the expression levels of hub genes in the Vizome database by heatmap **(A)** and gene-interaction plot **(B)**.

We verified the expression levels of hub genes in the Vizome database. The heatmap demonstrated that 18 hub genes showed high expression in the samples from the database (Figure 8A). In addition, four gene interactions of the hub genes were shown in Figure 8B. Furthermore, the expression level of hub genes in overall survival was shown in Supplementary Figure 2.

## Discussion

In recent years, since the rapid development of immunological checkpoint therapy such as CTLA-4 (4) and PD-1 (5) in AML, TME has attracted more and more attention as a key cell environment for immune cells, extracellular matrix molecules, and stromal cells (6, 7). Immunotherapy for cancer destroys cancer cells and destroys the immune system. There is increasing evidence that the key mechanism of interaction between the immune system and AML is the immune checkpoint in immune dynamics (34). Immune checkpoint, which is defined as co-stimulatory and co-suppressor molecules that regulate immune cell activity, could be coordinated as a regulatory loop to self-tolerate the immune system under normal physiological conditions (35, 36).

In our current study, we calculated the immune score, stromal score, and ESTIMATE score for each AML sample extracted from the TCGA database by applying the ESTIMATE algorithm. The results show that the immune scores for malignant tumor cases are statistically higher and are associated with worse survival outcomes, advanced tumor grades, and higher pathological stages. ESTIMATE algorithm-derived immune score was first calculated in AML to assess prognostic value and provide additional evidence for the biological basis of immunotherapy. In our research, the PPI network was built using the SRING tool and Cytoscape software. Finally, 18 TME-related hub genes were selected and the potential pathways such as immune response, inflammatory response, plasma membrane, and receptor activity were identified. We explored the associations between hub genes with immune infiltration in AML TME by using the deconvolution algorithm based on the TIMER database. We found that 18 hub genes including ITGAM, TNFRSF1B, HLA-DRB1, HLA-DRB5, and CX3CR1 were related to hematopoietic cell lineage, intestinal immune network for IgA production, natural killer cell mediated cytotoxicity, NOD like receptor signaling pathway, T cell receptor signaling pathway, and Toll like receptor signaling pathway.

Integrin alpha M (ITGAM) is a cell surface receptor selectively expressed on leukocytes (37), also known as differentiation 11b (CD11b), macrophage-1 antigen alpha subunit or macrophage receptor 1 alpha subunit (MAC1a), complement component 3 receptor alpha chain (CR3a). In the GENE database of the National Center for Biotechnology Information (NCBI), the protein is also named as systemic lupus erythematosus type 6 (SLEB6) or MO1A (38–40). It is a protein subunit that forms the heterodimeric integrin alpha-M beta-2 molecule with cluster of differentiation 18 (CD18), also known as complement receptor 3 (CR3) or MO1, macrophage-1 antigen or macrophage-1 antigen (Mac-1) (38–40). This protein is involved in cell activation, chemotaxis, cytotoxicity, phagocytosis, and regulates the interaction between leukemic cells and microenvironment by binding to its ligand, such as deactivated complement component 3b (iC3b), intercellular adhesion molecule (ICAM), fibrinogen, beta-glucan, coagulation factor X, etc. (41–46). More recently, ITGAM has also been defined as a marker for myeloid suppressor cells, which has been reported to be used by malignant cells to suppress anti-tumor immunity and promote malignant expansion or refractory therapy (47–49). Therefore, it can be speculated that ITGAM may be involved in the regulation of malignant AML cell biology, and its expression level may affect the prognosis of AML patients. Recently, a meta-analysis included 13 studies with a total of 2,619 patients (37). Results of the meta-analysis showed that ITGAM positivity was associated with lower complete remission rate (OR = 0.44; 95% CI, 0.25–0.79; *p* = 0.006) and shorter OS (HR = 0.66; 95% CI, 0.55–0.80; *p* < 0.0001), Consistent with our analysis, ITGAM positivity predicts a poor prognosis of AML patients. Therefore, ITGAM expression level might be considered a prognostic biomarker for AML patients.

TNF receptor superfamily member 1B (TNFRSF1B) is one of type I transmembrane receptors, which is also named as CD120b, TBPII, TNF-R-II, TNF-R75, TNFBR, TNFR1B, TNFR2, TNFR80, p75, p75TNFR (50). TNFRSF1B promotes tumor progression by maintaining a pro-tumor immune-microenvironment or by promoting the proliferation and survival of malignant cells. In the tumor microenvironment, TNFRSF1B is widely expressed in many types of cells, including immune cells and malignant cells (51). TNFRSF1B usually accelerates the malignant transformation and growth of tumor cells, rather than inducing cell death through apoptosis (52). Similar to tumor cells, TNFRSF1B protects immunosuppressive regulatory T (T_reg_) cells and myeloid-derived suppressor cells (MDSC) from the death-inducing TNF and thus enhances the proliferation and function of those tumor-promoting cells (53). To make matters worse, TNFRSF1B worsens the programmed death of phagocytic macrophages responsible for clearing of tumor cells. Mediating those direct and indirect effects, TNFRSF1B exacerbates cancer progression (54).

TNFRSF1B is mainly expressed on malignant cells and in the immunosuppressive cell compartment within the tumor microenvironment. It is involved in promoting tumor development and facilitated metastasis (50). Therefore, TNFRSF1B represents an attractive target for tumor therapy. Specifically, blocking the ligand TNF is an option. As TNFRSF1B is more highly expressed than TNFR1 in tumors and tumor-related cells, TNF is likely to have a tumor-promoting function instead of an inhibitory impact. TNF ablation effectively reduces tumor growth (55). In preclinical studies, the use of TNFRSF1B^+^ T_reg_ cells enhanced the efficacy of chemotherapy (56). In a clinical trial of patients with AML, patients received the demethylating agent, azacitidine, and the histone deacetylase inhibitor, panobinostat, which effectively eliminated TNFRSF1B^+^ T_reg_ cells in peripheral blood and bone marrow (57). These TNFRSF1B^+^ T_reg_ cells were earlier found as potent suppressive immune cell subset with enhanced migratory ability that promote disease progression and hamper tumor therapy (57, 58). Beneficial clinical responses are derived from more active effector T cells, as determined by increased production of interferon-γ and IL-2. Immunosuppressive microenvironment is the main obstacle to tumor therapy. In the past decade, immunotherapy using checkpoint closures and engineered t-cells has been a huge success (10). TNF is abundant in any tumor microenvironment. Tumor cells with high expression of TNFRSF1B resist TNF-induced cell death by ligand binding to TNFRSF1B. TNFRSF1B is highly expressed not only in tumor cells but also in immunosuppressive cells, including MDSC and T_reg_ cells (54–59). Therefore, TNFRSF1B is closely related to the immunosuppression ability of tumor promoting cells. All of these characteristics make TNFRSF1B an ideal candidate for targeted cancer therapy. Several studies (56, 57, 60) targeting TNFRSF1B already proved its great potential in tumor treating. Future investigations will provide more detailed knowledge about all facets and on the cell-type dependency of TNFRSF1B's immunosuppressive effects that we need to translate it into the treatment of malignant diseases.

Notably, the risk model was calculated based on 18 hub prognostic genes associated with AML TME. The AUC of the ROC curve reveals satisfactory prediction efficiency of risk signature. This new TME central gene-related risk scoring model provides a new theoretical basis for the prognosis assessment of AML patients, and is expected to be further applied in future clinical management. Immunotherapy is one of the most expensive cancer treatment groups and it is not clear how patient and disease-specific characteristics should guide the selection of immunotherapy. In addition, questions remain about their use in treatment, support and maintenance environments. In order to establish the role of immune-based therapy in managing highly heterogeneous diseases such as AML, costly and large, randomized trials are needed which requires the identification of adequate biomarkers to help predict treatment response and toxicities, and to accurately select patients for accrual (10). In our current work, we focused on genes characteristic of microenvironment, which in turn affect the development of AML and hence contribute to patients' overall survival. Our results may provide additional data in decoding the complex interaction of tumor, immunotherapies and tumor environment in AML.

It is important to note that limitations existed in our current study. Firstly, we only selected target data from the TCGA public database through biological algorithm approaches. We should validate the results of this article in clinical patients in further study. Secondly, 18 hub genes related to immune cells infiltration should be further studied to clarify the regulatory mechanism in immune infiltrates of AML. Finally, considering the choice of analytical approaches, we included a limited database for the screening of hub genes in the immune ecosystem, which may lead to biased results due to the neglect of other databases.

In summary, TME-related hub genes were identified from functional enrichment analysis of TCGA database based on ESTIMATE algorithm. We believed that these hub genes might become potential biomarkers of AML according to survival analysis and prognostic value evaluation. In addition, RS provided a novel theoretical basis for predicting survival conditions of AML patients. Finally, further investigation of TME-related hub genes might contribute to new insights into the potential association of TME with AML prognosis in a synthetical way.

## Conclusion

In our study, we selected the transcriptional profiles from public databases based on bioinformatic algorithm and identified specific signatures associated with matrix and immune cell infiltration in AML TME.

## Data Availability Statement

The datasets analyzed in this study can be found in The Cancer Genome Atlas (https://portal.gdc.cancer.gov/) (Level 3 gene transcriptome profiles of AML patients).

## Author Contributions

JN, SL, and YZ designed the study and analyzed the data. FQ, JF, and YW drafted the article. SY was responsible for language correction. All authors finally approved the paper.

### Conflict of Interest

The authors declare that the research was conducted in the absence of any commercial or financial relationships that could be construed as a potential conflict of interest.

## Supplementary Material

Supplementary Figure 1 The heatmap and volcano plot based on stromal scores. In stromal score group, heatmap **(A)** and volcano plot **(B)** were used to demonstrated differential expressed genes.

Supplementary Figure 2 Overall survival analysis in Vizome database. The expression level of 18 hub genes were exhibited by overall survival.

Supplementary Figure 3 The flow diagram representing the work. The flow diagram representing the work was shown. AML, Acute myeloid leukemia; TCGA, the Cancer Genome Atlas; CALGB, Cancer and Acute Leukemia Group B; PPI, Protein-protein interaction; GSEA, gene set enrichment analysis.

Supplementary Table 1 Immune scores, stromal scores, and ESTIMATE scores of AML patients.

Supplementary Table 2 DEGs based on immune score group.

Supplementary Table 3 DEGs based on stromal score group.

## References

[1] Ferrara F Schiffer CA. Acute myeloid leukaemia in adults. Lancet. (2013) 381:484–95. 10.1016/S0140-6736(12)61727-9 23399072

[2] Dohner H Estey E Grimwade D Amadori S Appelbaum FR Buchner T. Diagnosis and management of AML in adults: 2017 ELN recommendations from an international expert panel. Blood. (2017) 129:424–47. 10.1182/blood-2016-08-733196 27895058 PMC5291965

[3] Yang D Zhang X Zhang X Xu Y. The progress and current status of immunotherapy in acute myeloid leukemia. Ann Hematol. (2017) 96:1965–82. 10.1007/s00277-017-3148-x 29080982

[4] Davids MS Kim HT Bachireddy P Costello C Liguori R Savell A. Ipilimumab for patients with relapse after allogeneic transplantation. N Engl J Med. (2016) 375:143–53. 10.1056/NEJMoa1601202 27410923 PMC5149459

[5] Sehgal A Whiteside TL Boyiadzis M. Programmed death-1 checkpoint blockade in acute myeloid leukemia. Exp Opin Biol Ther. (2015) 15:1191–203. 10.1517/14712598.2015.1051028 26036819 PMC4778424

[6] Escudier B Sharma P McDermott DF George S Hammers HJ Srinivas S. CheckMate 025 randomized phase 3 study: outcomes by key baseline factors and prior therapy for nivolumab versus everolimus in advanced renal cell carcinoma. Euro Urol. (2017) 72:962–71. 10.1016/j.eururo.2017.02.010 28262413

[7] Wu T Dai Y. Tumor microenvironment and therapeutic response. Cancer Lett. (2017) 387:61–8. 10.1016/j.canlet.2016.01.043 26845449

[8] Hanahan D Weinberg RA. The hallmarks of cancer. Cell. (2000) 100:57–70. 10.1016/S0092-8674(00)81683-9 10647931

[9] Hanahan D Coussens LM. Accessories to the crime: functions of cells recruited to the tumor microenvironment. Cancer Cell. (2012) 21:309–22. 10.1016/j.ccr.2012.02.022 22439926

[10] Gbolahan OB Zeidan AM Stahl M Abu Zaid M Farag S Paczesny S. Immunotherapeutic concepts to target acute myeloid leukemia: focusing on the role of monoclonal antibodies, hypomethylating agents and the leukemic microenvironment. Int J Mol Sci. (2017) 18:1660. 10.3390/ijms18081660 28758974 PMC5578050

[11] Curry JM Sprandio J Cognetti D Luginbuhl A Bar-ad V Pribitkin E. Tumor microenvironment in head and neck squamous cell carcinoma. Semin Oncol. (2014) 41:217–34. 10.1053/j.seminoncol.2014.03.003 24787294

[12] Cooper LA Gutman DA Chisolm C Appin C Kong J Rong Y. The tumor microenvironment strongly impacts master transcriptional regulators and gene expression class of glioblastoma. Am J Pathol. (2012) 180:2108–19. 10.1016/j.ajpath.2012.01.040 22440258 PMC3354586

[13] Galon J Pages F Marincola FM Thurin M Trinchieri G Fox BA. The immune score as a new possible approach for the classification of cancer. J Transl Med. (2012) 10:1. 10.1186/1479-5876-10-1 22214470 PMC3269368

[14] Yoshihara K Shahmoradgoli M Martinez E Vegesna R Kim H Torres-Garcia W. Inferring tumour purity and stromal and immune cell admixture from expression data. Nat Commun. (2013) 4:2612. 10.1038/ncomms3612 24113773 PMC3826632

[15] Senbabaoglu Y Gejman RS Winer AG Liu M Van Allen EM de Velasco G. Tumor immune microenvironment characterization in clear cell renal cell carcinoma identifies prognostic and immunotherapeutically relevant messenger RNA signatures. Genome Biol. (2016) 17:231. 10.1186/s13059-016-1092-z 27855702 PMC5114739

[16] Winslow S Lindquist KE Edsjo A Larsson C. The expression pattern of matrix-producing tumor stroma is of prognostic importance in breast cancer. BMC Cancer. (2016) 16:841. 10.1186/s12885-016-2864-2 27809802 PMC5095990

[17] Carter SL Cibulskis K Helman E McKenna A Shen H Zack T. Absolute quantification of somatic DNA alterations in human cancer. Nat Biotechnol. (2012) 30:413–21. 10.1038/nbt.2203 22544022 PMC4383288

[18] Aldape K Zadeh G Mansouri S Reifenberger G von Deimling A. Glioblastoma: pathology, molecular mechanisms and markers. Acta Neuropathol. (2015) 129:829–48. 10.1007/s00401-015-1432-1 25943888

[19] Cancer Genome Atlas Research N Comprehensive genomic characterization defines human glioblastoma genes and core pathways. Nature. (2008) 455:1061–8. 10.1038/nature07385 18772890 PMC2671642

[20] Shah N Wang P Wongvipat J Karthaus WR Abida W Armenia J. Regulation of the glucocorticoid receptor via a BET-dependent enhancer drives antiandrogen resistance in prostate cancer. eLife. (2017) 6:e27861. 10.7554/eLife.27861.019 28891793 PMC5593504

[21] Priedigkeit N Watters RJ Lucas PC Basudan A Bhargava R Horne W. Exome-capture RNA sequencing of decade-old breast cancers and matched decalcified bone metastases. JCI Insight. (2017) 2:e95703. 10.1172/jci.insight.95703 28878133 PMC5621874

[22] Alonso MH Ausso S Lopez-Doriga A Cordero D Guino E Sole X. Comprehensive analysis of copy number aberrations in microsatellite stable colon cancer in view of stromal component. Br J Cancer. (2017) 117:421–31. 10.1038/bjc.2017.208 28683472 PMC5537504

[23] Ritchie ME Phipson B Wu D Hu Y Law CW Shi W. limma powers differential expression analyses for RNA-sequencing and microarray studies. Nucleic Acids Res. (2015) 43:e47. 10.1093/nar/gkv007 25605792 PMC4402510

[24] Aalen OO. A linear regression model for the analysis of life times. Stat Med. (1989) 8:907–25. 10.1002/sim.4780080803 2678347

[25] Aalen OO. Further results on the non-parametric linear regression model in survival analysis. Stat Med. (1993) 12:1569–88. 10.1002/sim.4780121705 8235179

[26] Chen H Boutros PC. VennDiagram: a package for the generation of highly-customizable Venn and Euler diagrams in R. BMC Bioinformatics. (2011) 12:35. 10.1186/1471-2105-12-35 21269502 PMC3041657

[27] Kanehisa M Goto S. KEGG: kyoto encyclopedia of genes and genomes. Nucleic Acids Res. (2000) 28:27–30. 10.1093/nar/28.1.27 10592173 PMC102409

[28] Tilford CA Siemers NO. Gene set enrichment analysis. Methods Mol Biol. (2009) 563:99–121. 10.1007/978-1-60761-175-2_6 19597782

[29] Szklarczyk D Franceschini A Wyder S Forslund K Heller D Huerta-Cepas J. STRING v10: protein-protein interaction networks, integrated over the tree of life. Nucleic Acids Res. (2015) 43:D447–52. 10.1093/nar/gku1003 25352553 PMC4383874

[30] Shannon P Markiel A Ozier O Baliga NS Wang JT Ramage D. Cytoscape: a software environment for integrated models of biomolecular interaction networks. Genome Res. (2003) 13:2498–504. 10.1101/gr.1239303 14597658 PMC403769

[31] Chin CH Chen SH Wu HH Ho CW Ko MT Lin CY. cytoHubba: identifying hub objects and sub-networks from complex interactome. BMC Syst Biol. (2014) 8(Suppl 4):S11. 10.1186/1752-0509-8-S4-S11 25521941 PMC4290687

[32] Heagerty PJ Lumley T Pepe MS. Time-dependent ROC curves for censored survival data and a diagnostic marker. Biometrics. (2000) 56:337–44. 10.1111/j.0006-341X.2000.00337.x 10877287

[33] Tyner JW Tognon CE Bottomly D Wilmot B Kurtz SE Savage SL. Functional genomic landscape of acute myeloid leukaemia. Nature. (2018) 562:526–31. 10.1038/s41586-018-0623-z 30333627 PMC6280667

[34] Maiga A Lemieux S Pabst C Lavallee VP Bouvier M Sauvageau G. Transcriptome analysis of G protein-coupled receptors in distinct genetic subgroups of acute myeloid leukemia: identification of potential disease-specific targets. Blood Cancer J. (2016) 6:e431. 10.1038/bcj.2016.36 27258612 PMC5141352

[35] Driessens G Kline J Gajewski TF. Costimulatory and coinhibitory receptors in anti-tumor immunity. Immunol Rev. (2009) 229:126–44. 10.1111/j.1600-065X.2009.00771.x 19426219 PMC3278040

[36] Syn NL Teng MWL Mok TSK Soo RA. *De-novo* and acquired resistance to immune checkpoint targeting. Lancet Oncol. (2017) 18:e731–41. 10.1016/S1470-2045(17)30607-1 29208439

[37] Xu S Li X Zhang J Chen J. Prognostic value of CD11b expression level for acute myeloid leukemia patients: a meta-analysis. PLoS ONE. (2015) 10:e0135981. 10.1371/journal.pone.0135981 26309131 PMC4550244

[38] Hickstein DD Ozols J Williams SA Baenziger JU Locksley RM Roth GJ. Isolation and characterization of the receptor on human neutrophils that mediates cellular adherence. J Biol Chem. (1987) 262:5576–80. 3553180

[39] Arnaout MA Gupta SK Pierce MW Tenen DG. Amino acid sequence of the alpha subunit of human leukocyte adhesion receptor Mo1 (complement receptor type 3). J Cell Biol. (1988) 106:2153–8. 10.1083/jcb.106.6.2153 2454931 PMC2115146

[40] Arnaout MA Lanier LL Faller DV. Relative contribution of the leukocyte molecules Mo1, LFA-1, and p150,95 (LeuM5) in adhesion of granulocytes and monocytes to vascular endothelium is tissue- and stimulus-specific. J Cell Physiol. (1988) 137:305–9. 10.1002/jcp.1041370214 3056960

[41] Fan ST Edgington TS. Coupling of the adhesive receptor CD11b/CD18 to functional enhancement of effector macrophage tissue factor response. J Clin Invest. (1991) 87:50–7. 10.1172/JCI115000 1670636 PMC294989

[42] Coombe DR Watt SM Parish CR. Mac-1 (CD11b/CD18) and CD45 mediate the adhesion of hematopoietic progenitor cells to stromal cell elements via recognition of stromal heparan sulfate. Blood. (1994) 84:739–52. 10.1182/blood.V84.3.739.bloodjournal843739 8043862

[43] Kusunoki T Tsuruta S Higashi H Hosoi S Hata D Sugie K. Involvement of CD11b/CD18 in enhanced neutrophil adhesion by Fc gamma receptor stimulation. J Leukoc Biol. (1994) 55:735–42. 10.1002/jlb.55.6.735 7910840

[44] Simon DI Ezratty AM Francis SA Rennke H Loscalzo J. Fibrin(ogen) is internalized and degraded by activated human monocytoid cells via Mac-1 (CD11b/CD18): a nonplasmin fibrinolytic pathway. Blood. (1993) 82:2414–22. 10.1182/blood.V82.8.2414.2414 8400291

[45] Ueda T Rieu P Brayer J Arnaout MA. Identification of the complement iC3b binding site in the beta 2 integrin CR3 (CD11b/CD18). Proc Natl Acad Sci USA. (1994) 91:10680–4. 10.1073/pnas.91.22.10680 7524101 PMC45085

[46] Lee JO Rieu P Arnaout MA Liddington R. Crystal structure of the A domain from the alpha subunit of integrin CR3 (CD11b/CD18). Cell. (1995) 80:631–8. 10.1016/0092-8674(95)90517-0 7867070

[47] Shojaei F Wu X Malik AK Zhong C Baldwin ME Schanz S. Tumor refractoriness to anti-VEGF treatment is mediated by CD11b+Gr1+ myeloid cells. Nat Biotechnol. (2007) 25:911–20. 10.1038/nbt1323 17664940

[48] De Veirman K Van Valckenborgh E Lahmar Q Geeraerts X De Bruyne E Menu E. Myeloid-derived suppressor cells as therapeutic target in hematological malignancies. Front Oncol. (2014) 4:349. 10.3389/fonc.2014.00349 25538893 PMC4258607

[49] Younos IH Abe F Talmadge JE. Myeloid-derived suppressor cells: their role in the pathophysiology of hematologic malignancies and potential as therapeutic targets. Leuk Lymphoma. (2015) 56:2251–63. 10.3109/10428194.2014.987141 25407654

[50] Sheng Y Li F Qin Z. TNF receptor 2 makes tumor necrosis factor a friend of tumors. Front Immunol. (2018) 9:1170. 10.3389/fimmu.2018.01170 29892300 PMC5985372

[51] Purdue MP Hofmann JN Kemp TJ Chaturvedi AK Lan Q Park JH. A prospective study of 67 serum immune and inflammation markers and risk of non-Hodgkin lymphoma. Blood. (2013) 122:951–7. 10.1182/blood-2013-01-481077 23814017 PMC3739038

[52] Babic A Shah SM Song M Wu K Meyerhardt JA Ogino S. Soluble tumour necrosis factor receptor type II and survival in colorectal cancer. Br J Cancer. (2016) 114:995–1002. 10.1038/bjc.2016.85 27031855 PMC4984918

[53] Zhang T Jiao J Jiao X Zhao L Tian X Zhang Q. Aberrant frequency of TNFR2(+) Treg and related cytokines in patients with CIN and cervical cancer. Oncotarget. (2018) 9:5073–83. 10.18632/oncotarget.23581 29435163 PMC5797034

[54] Vanamee ES Faustman DL. TNFR2: a novel target for cancer immunotherapy. Trends Mol Med. (2017) 23:1037–46. 10.1016/j.molmed.2017.09.007 29032004

[55] Atretkhany KS Nosenko MA Gogoleva VS Zvartsev RV Qin Z Nedospasov SA. TNF neutralization results in the delay of transplantable tumor growth and reduced MDSC accumulation. Front Immunol. (2016) 7:147. 10.3389/fimmu.2016.00147 27148266 PMC4835443

[56] van der Most RG Currie AJ Mahendran S Prosser A Darabi A Robinson BW. Tumor eradication after cyclophosphamide depends on concurrent depletion of regulatory T cells: a role for cycling TNFR2-expressing effector-suppressor T cells in limiting effective chemotherapy. Cancer Immunol Immunother. (2009) 58:1219–28. 10.1007/s00262-008-0628-9 19052741 PMC11030690

[57] Govindaraj C Tan P Walker P Wei A Spencer A Plebanski M. Reducing TNF receptor 2+ regulatory T cells via the combined action of azacitidine and the HDAC inhibitor, panobinostat for clinical benefit in acute myeloid leukemia patients. Clin Cancer Res. (2014) 20:724–35. 10.1158/1078-0432.CCR-13-1576 24297862

[58] Chen X Baumel M Mannel DN Howard OM Oppenheim JJ. Interaction of TNF with TNF receptor type 2 promotes expansion and function of mouse CD4+CD25+ T regulatory cells. J Immunol. (2007) 179:154–61. 10.4049/jimmunol.179.1.154 17579033

[59] Sade-Feldman M Kanterman J Ish-Shalom E Elnekave M Horwitz E Baniyash M. Tumor necrosis factor-α blocks differentiation and enhances suppressive activity of immature myeloid cells during chronic inflammation. Immunity. (2013) 38:541–54. 10.1016/j.immuni.2013.02.007 23477736

[60] Govindaraj C Madondo M Kong YY Tan P Wei A Plebanski M. Lenalidomide-based maintenance therapy reduces TNF receptor 2 on CD4 T cells and enhances immune effector function in acute myeloid leukemia patients. Am J Hematol. (2014) 89:795–802. 10.1002/ajh.23746 24757092

