# Supplementary figures and images for: Screening the Cancer Genome Atlas Database for Genes of Prognostic Value in Acute Myeloid Leukemia
Source: Front Oncol. 2020 Jan 21;9:1509. doi: 10.3389/fonc.2019.01509 (PMC6990132; doi:10.3389/fonc.2019.01509)

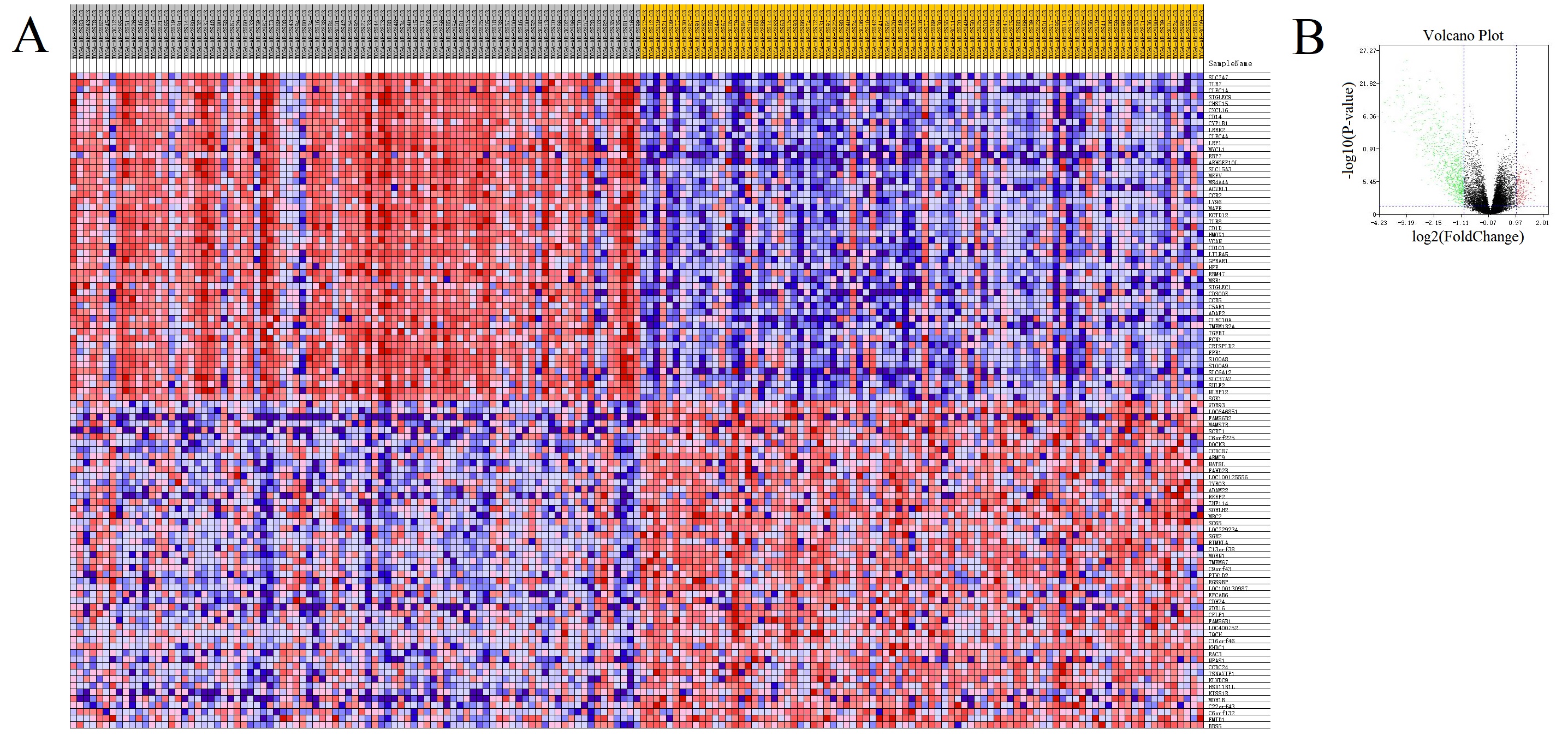

Supplement: Supplementary Figure 1 — The heatmap and volcano plot based on stromal scores. In stromal score group, heatmap (A) and volcano plot (B) were used to demonstrated differential expressed genes. [file Image_1.PNG]

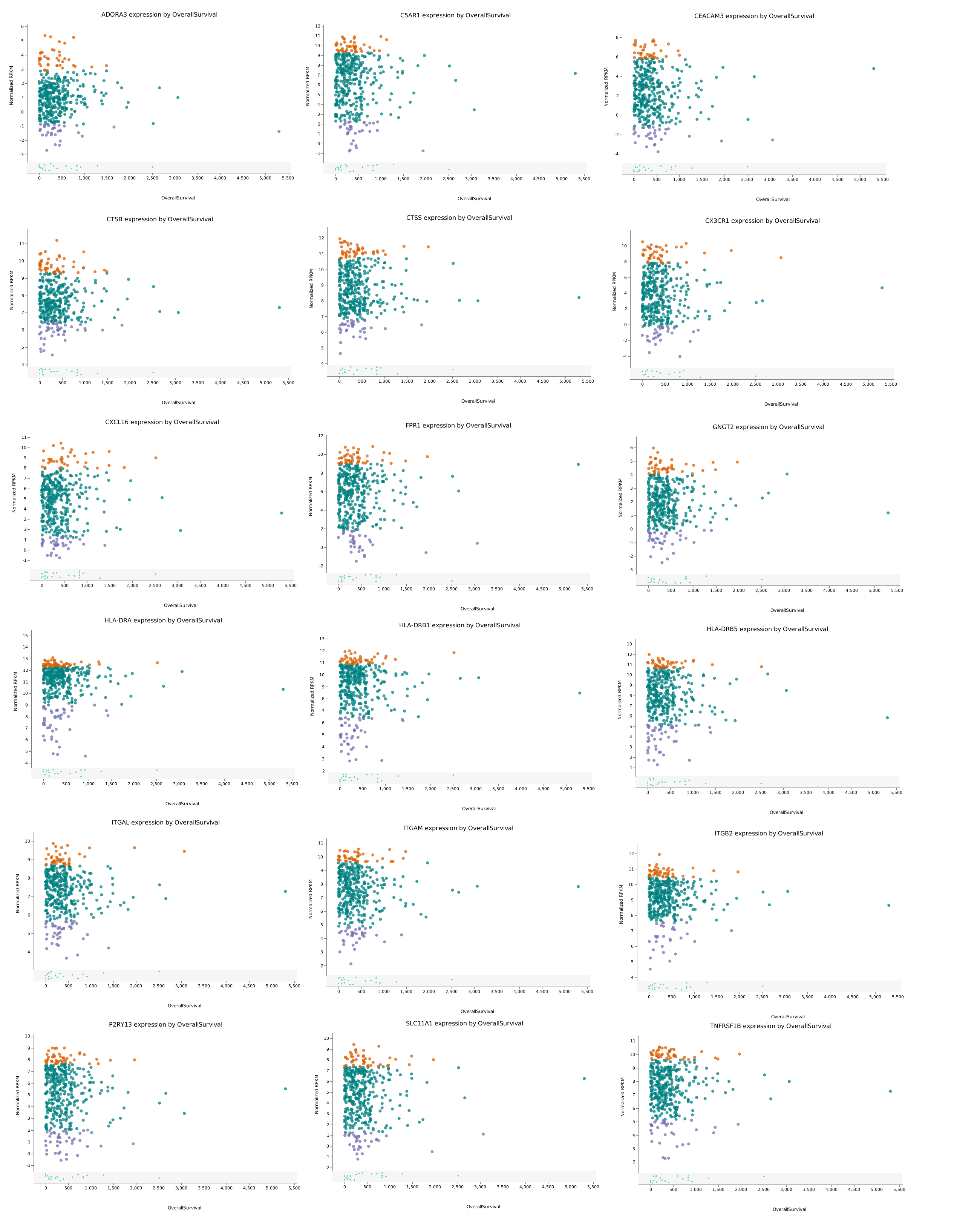

Supplement: Supplementary Figure 2 — Overall survival analysis in Vizome database. The expression level of 18 hub genes were exhibited by overall survival. [file Image_2.PNG]

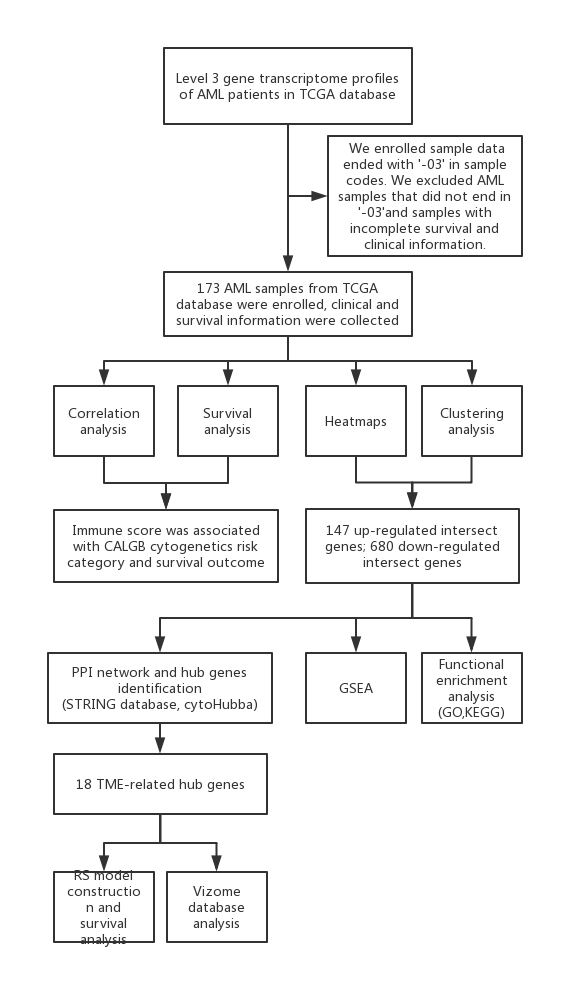

Supplement: Supplementary Figure 3 — The flow diagram representing the work. The flow diagram representing the work was shown. AML, Acute myeloid leukemia; TCGA, the Cancer Genome Atlas; CALGB, Cancer and Acute Leukemia Group B; PPI, Protein-protein interaction; GSEA, gene set enrichment analysis. [file Image_3.PNG]
