# Supplementary material for: Screening the Cancer Genome Atlas Database for Genes of Prognostic Value in Acute Myeloid Leukemia
Source: Front Oncol. 2020 Jan 21;9:1509. doi: 10.3389/fonc.2019.01509 (PMC6990132; doi:10.3389/fonc.2019.01509)
Supplement: Supplementary Table 1 — Immune scores, stromal scores, and ESTIMATE scores of AML patients. [file Table_1.DOCX]

| Sample ID | Stromal score | Immune score | ESTIMATE score |
| --- | --- | --- | --- |
| TCGA-AB-2803-03 | -957.59 | 2098.98 | 1141.39 |
| TCGA-AB-2805-03 | -705.43 | 3050.71 | 2345.27 |
| TCGA-AB-2806-03 | -1171.75 | 1678.63 | 506.89 |
| TCGA-AB-2807-03 | -834.36 | 2260.22 | 1425.86 |
| TCGA-AB-2808-03 | -845.56 | 2670.19 | 1824.64 |
| TCGA-AB-2810-03 | -1735.4 | 2510.82 | 775.42 |
| TCGA-AB-2811-03 | -1249.43 | 3093.47 | 1844.03 |
| TCGA-AB-2812-03 | -1472.83 | 1864.89 | 392.06 |
| TCGA-AB-2813-03 | -352.55 | 3282.7 | 2930.14 |
| TCGA-AB-2814-03 | -1452.73 | 2116.58 | 663.85 |
| TCGA-AB-2815-03 | -425.53 | 3081.63 | 2656.1 |
| TCGA-AB-2816-03 | -748.92 | 3447.06 | 2698.14 |
| TCGA-AB-2817-03 | -722.44 | 2945.13 | 2222.69 |
| TCGA-AB-2818-03 | -755.64 | 3112.34 | 2356.7 |
| TCGA-AB-2819-03 | -971.98 | 1990.48 | 1018.5 |
| TCGA-AB-2820-03 | -1350.03 | 2489.67 | 1139.64 |
| TCGA-AB-2821-03 | -806.38 | 2642.07 | 1835.69 |
| TCGA-AB-2822-03 | -1373.31 | 1763.23 | 389.92 |
| TCGA-AB-2823-03 | -1277.38 | 2053.21 | 775.84 |
| TCGA-AB-2824-03 | -1217.68 | 2619.54 | 1401.86 |
| TCGA-AB-2825-03 | -737.29 | 3089.92 | 2352.63 |
| TCGA-AB-2826-03 | -818.95 | 3222.02 | 2403.07 |
| TCGA-AB-2828-03 | -543.18 | 3022.84 | 2479.66 |
| TCGA-AB-2830-03 | -1136.61 | 2683.65 | 1547.04 |
| TCGA-AB-2832-03 | -1693.75 | 2425.04 | 731.29 |
| TCGA-AB-2833-03 | -554.95 | 3411.09 | 2856.14 |
| TCGA-AB-2834-03 | -795.81 | 2844.83 | 2049.01 |
| TCGA-AB-2835-03 | -810.89 | 3066.53 | 2255.64 |
| TCGA-AB-2836-03 | -534.09 | 3355.86 | 2821.77 |
| TCGA-AB-2837-03 | -317.3 | 3316.21 | 2998.9 |
| TCGA-AB-2838-03 | -454.89 | 3259.51 | 2804.63 |
| TCGA-AB-2839-03 | -1297.64 | 2093.82 | 796.18 |
| TCGA-AB-2840-03 | -1064.98 | 2115.48 | 1050.51 |
| TCGA-AB-2841-03 | -1349.07 | 2471.45 | 1122.38 |
| TCGA-AB-2842-03 | -1070.24 | 2652.16 | 1581.91 |
| TCGA-AB-2843-03 | -754.82 | 3116.63 | 2361.81 |
| TCGA-AB-2844-03 | -990.1 | 2680.13 | 1690.03 |
| TCGA-AB-2845-03 | -968.84 | 2799.66 | 1830.81 |
| TCGA-AB-2846-03 | -603.65 | 3013.74 | 2410.08 |
| TCGA-AB-2847-03 | -1526.55 | 2657.57 | 1131.01 |
| TCGA-AB-2848-03 | -900 | 3076.78 | 2176.77 |
| TCGA-AB-2849-03 | -1133.71 | 1606.38 | 472.67 |
| TCGA-AB-2851-03 | -755.61 | 3394.32 | 2638.72 |
| TCGA-AB-2853-03 | -1419.18 | 2174.88 | 755.71 |
| TCGA-AB-2854-03 | -713.22 | 3034.59 | 2321.37 |
| TCGA-AB-2855-03 | -1381.45 | 2068.34 | 686.89 |
| TCGA-AB-2856-03 | -952.2 | 3150.15 | 2197.95 |
| TCGA-AB-2857-03 | -494.19 | 2698.17 | 2203.98 |
| TCGA-AB-2858-03 | -1051.95 | 2207.23 | 1155.28 |
| TCGA-AB-2859-03 | -1163.95 | 2842.09 | 1678.14 |
| TCGA-AB-2860-03 | -562.26 | 3051.41 | 2489.15 |
| TCGA-AB-2861-03 | -1267.33 | 2389.18 | 1121.85 |
| TCGA-AB-2862-03 | -1305.23 | 2040.21 | 734.98 |
| TCGA-AB-2863-03 | -1233.12 | 1844.11 | 610.99 |
| TCGA-AB-2865-03 | -297.73 | 3148.23 | 2850.51 |
| TCGA-AB-2866-03 | -989.09 | 3230.53 | 2241.44 |
| TCGA-AB-2867-03 | -1109.48 | 3167.69 | 2058.21 |
| TCGA-AB-2868-03 | -1094.44 | 3145.41 | 2050.97 |
| TCGA-AB-2869-03 | -1452.13 | 2255.34 | 803.21 |
| TCGA-AB-2870-03 | -561.03 | 3181.32 | 2620.3 |
| TCGA-AB-2871-03 | -1377.73 | 2646.37 | 1268.65 |
| TCGA-AB-2872-03 | -1213.16 | 1941.83 | 728.67 |
| TCGA-AB-2873-03 | -317.69 | 3316.93 | 2999.24 |
| TCGA-AB-2874-03 | -1268.1 | 2285.68 | 1017.58 |
| TCGA-AB-2875-03 | -1303.36 | 2319.13 | 1015.77 |
| TCGA-AB-2877-03 | -1659.15 | 1607.95 | -51.2 |
| TCGA-AB-2879-03 | -1753.86 | 1329.53 | -424.33 |
| TCGA-AB-2880-03 | -1050.87 | 2835.4 | 1784.53 |
| TCGA-AB-2881-03 | -891.99 | 3188.92 | 2296.93 |
| TCGA-AB-2882-03 | -235.75 | 3352.15 | 3116.4 |
| TCGA-AB-2884-03 | -831.42 | 3078.7 | 2247.27 |
| TCGA-AB-2885-03 | -1348.06 | 2353.63 | 1005.57 |
| TCGA-AB-2886-03 | -1155.08 | 1909.24 | 754.16 |
| TCGA-AB-2887-03 | -1385.62 | 2082.27 | 696.65 |
| TCGA-AB-2888-03 | -490.76 | 3758.87 | 3268.12 |
| TCGA-AB-2889-03 | -766.26 | 2956.85 | 2190.59 |
| TCGA-AB-2890-03 | -716.46 | 3481.5 | 2765.04 |
| TCGA-AB-2891-03 | -933.06 | 2923.85 | 1990.78 |
| TCGA-AB-2895-03 | -1470.38 | 1839.34 | 368.96 |
| TCGA-AB-2896-03 | -1180.21 | 2534.1 | 1353.9 |
| TCGA-AB-2897-03 | -1220.01 | 1967.8 | 747.8 |
| TCGA-AB-2898-03 | -1216.26 | 2598.21 | 1381.95 |
| TCGA-AB-2899-03 | -638.92 | 3039.91 | 2400.99 |
| TCGA-AB-2900-03 | -957.98 | 2861.97 | 1904 |
| TCGA-AB-2901-03 | -1259.53 | 1753.66 | 494.13 |
| TCGA-AB-2903-03 | -1396.73 | 1579.75 | 183.02 |
| TCGA-AB-2904-03 | -1122.03 | 2273.84 | 1151.81 |
| TCGA-AB-2908-03 | -1059.53 | 2935.57 | 1876.04 |
| TCGA-AB-2909-03 | -1462.53 | 2150.49 | 687.96 |
| TCGA-AB-2910-03 | -1549.35 | 2388.6 | 839.25 |
| TCGA-AB-2911-03 | -630.93 | 3391.72 | 2760.79 |
| TCGA-AB-2912-03 | -652.24 | 2662.44 | 2010.2 |
| TCGA-AB-2913-03 | -1534.51 | 1799.54 | 265.03 |
| TCGA-AB-2914-03 | -885.1 | 2756.55 | 1871.45 |
| TCGA-AB-2915-03 | -840.98 | 3268.52 | 2427.54 |
| TCGA-AB-2916-03 | -448.3 | 3648.38 | 3200.08 |
| TCGA-AB-2917-03 | -1279.81 | 2526.91 | 1247.1 |
| TCGA-AB-2918-03 | -1238.23 | 2501.17 | 1262.95 |
| TCGA-AB-2919-03 | -1641.15 | 1368.53 | -272.62 |
| TCGA-AB-2920-03 | -1660.43 | 2343.93 | 683.5 |
| TCGA-AB-2921-03 | -1094.73 | 3000.09 | 1905.36 |
| TCGA-AB-2924-03 | -1099.34 | 3030.97 | 1931.63 |
| TCGA-AB-2925-03 | -1094.3 | 2864.97 | 1770.67 |
| TCGA-AB-2927-03 | -611.38 | 3326.88 | 2715.5 |
| TCGA-AB-2928-03 | -1888.81 | 1798.47 | -90.34 |
| TCGA-AB-2929-03 | -1058.12 | 2977.65 | 1919.53 |
| TCGA-AB-2930-03 | -1272.53 | 2524.53 | 1251.99 |
| TCGA-AB-2931-03 | -1376.55 | 2179.1 | 802.54 |
| TCGA-AB-2932-03 | -791.81 | 3224.63 | 2432.82 |
| TCGA-AB-2933-03 | -794.34 | 3274.8 | 2480.46 |
| TCGA-AB-2934-03 | -683.74 | 3432.53 | 2748.8 |
| TCGA-AB-2935-03 | -653.62 | 3374.03 | 2720.41 |
| TCGA-AB-2936-03 | -1165.61 | 2738.4 | 1572.79 |
| TCGA-AB-2937-03 | -1294.37 | 2212.27 | 917.9 |
| TCGA-AB-2938-03 | -207.13 | 2177.21 | 1970.08 |
| TCGA-AB-2939-03 | -1159.6 | 2467.86 | 1308.26 |
| TCGA-AB-2940-03 | -883.48 | 2860.39 | 1976.9 |
| TCGA-AB-2941-03 | -1398.47 | 1625.8 | 227.33 |
| TCGA-AB-2942-03 | -840.39 | 3106.54 | 2266.15 |
| TCGA-AB-2943-03 | -1042.38 | 3163.32 | 2120.93 |
| TCGA-AB-2944-03 | -1427.82 | 2468.6 | 1040.79 |
| TCGA-AB-2946-03 | -805.01 | 3051.62 | 2246.62 |
| TCGA-AB-2948-03 | -800.31 | 3260.43 | 2460.12 |
| TCGA-AB-2949-03 | -1076.92 | 2616.32 | 1539.4 |
| TCGA-AB-2950-03 | -1139.82 | 1637.44 | 497.62 |
| TCGA-AB-2952-03 | -803.9 | 3347.81 | 2543.91 |
| TCGA-AB-2954-03 | -781.55 | 3036.07 | 2254.52 |
| TCGA-AB-2955-03 | -1295.46 | 2369.05 | 1073.6 |
| TCGA-AB-2956-03 | -1313.47 | 2789.24 | 1475.78 |
| TCGA-AB-2959-03 | -997.48 | 2473.45 | 1475.97 |
| TCGA-AB-2963-03 | -1071.68 | 2543.62 | 1471.94 |
| TCGA-AB-2964-03 | -1274.69 | 1674.25 | 399.55 |
| TCGA-AB-2965-03 | -736.87 | 2785.74 | 2048.87 |
| TCGA-AB-2966-03 | -1468.67 | 2224.91 | 756.24 |
| TCGA-AB-2967-03 | -830.8 | 2880.83 | 2050.03 |
| TCGA-AB-2969-03 | -869.94 | 3159.53 | 2289.59 |
| TCGA-AB-2970-03 | -614.43 | 2739.26 | 2124.83 |
| TCGA-AB-2971-03 | -715.15 | 3309.19 | 2594.04 |
| TCGA-AB-2972-03 | -1726.73 | 1651.04 | -75.68 |
| TCGA-AB-2973-03 | -455.71 | 3632.61 | 3176.89 |
| TCGA-AB-2975-03 | -1487.56 | 2282.66 | 795.1 |
| TCGA-AB-2976-03 | -1402.6 | 2059.98 | 657.39 |
| TCGA-AB-2977-03 | -1097.89 | 3302.6 | 2204.71 |
| TCGA-AB-2978-03 | -890.96 | 2947.2 | 2056.24 |
| TCGA-AB-2979-03 | -1117.78 | 2652.51 | 1534.73 |
| TCGA-AB-2980-03 | -972 | 1475.85 | 503.85 |
| TCGA-AB-2981-03 | -202.78 | 3619.74 | 3416.96 |
| TCGA-AB-2982-03 | -734.09 | 2304.41 | 1570.31 |
| TCGA-AB-2983-03 | -891.09 | 2683.02 | 1791.93 |
| TCGA-AB-2984-03 | -1517.81 | 1769.79 | 251.99 |
| TCGA-AB-2985-03 | -721.84 | 3620.93 | 2899.09 |
| TCGA-AB-2986-03 | -1576.29 | 2605.61 | 1029.32 |
| TCGA-AB-2987-03 | 435.75 | 3971.97 | 4407.72 |
| TCGA-AB-2988-03 | -1516.77 | 2211.93 | 695.15 |
| TCGA-AB-2990-03 | -1598.09 | 2247.53 | 649.45 |
| TCGA-AB-2991-03 | -1171.42 | 2172.37 | 1000.94 |
| TCGA-AB-2992-03 | -389.52 | 3229.37 | 2839.84 |
| TCGA-AB-2993-03 | -1231.99 | 1823.93 | 591.94 |
| TCGA-AB-2994-03 | -1062.43 | 2707.91 | 1645.48 |
| TCGA-AB-2995-03 | -1346.17 | 2301.07 | 954.9 |
| TCGA-AB-2996-03 | -693.21 | 2762.73 | 2069.52 |
| TCGA-AB-2998-03 | -1301.45 | 2189.05 | 887.6 |
| TCGA-AB-2999-03 | -1143.34 | 2186.34 | 1043 |
| TCGA-AB-3000-03 | -1010.38 | 2688.11 | 1677.73 |
| TCGA-AB-3001-03 | -818.56 | 2316.75 | 1498.18 |
| TCGA-AB-3002-03 | -1060.7 | 2609.09 | 1548.39 |
| TCGA-AB-3005-03 | -1262.44 | 2669.21 | 1406.78 |
| TCGA-AB-3006-03 | -1309.93 | 1797.52 | 487.59 |
| TCGA-AB-3007-03 | -1571.73 | 1734.11 | 162.38 |
| TCGA-AB-3008-03 | -920.98 | 2227.48 | 1306.5 |
| TCGA-AB-3009-03 | -1073.74 | 1979.06 | 905.32 |
| TCGA-AB-3011-03 | -1303.23 | 1732.15 | 428.92 |
| TCGA-AB-3012-03 | -692.25 | 1842.87 | 1150.63 |
